# Fabry pedigree analysis: A successful program for targeted genetic approach

**DOI:** 10.1002/mgg3.794

**Published:** 2019-06-06

**Authors:** Paula A. Rozenfeld, Francisca M. Masllorens, Norma Roa, Fernanda Rodriguez, Mariela Bonnano, Carolina Yvorra, Romina Ceci

**Affiliations:** ^1^ Departamento de Ciencias Biologicas, CONICET, Facultad de Ciencias Exactas IIFP, Universidad Nacional de La Plata La Plata Argentina; ^2^ Hospital Posadas Buenos Aires Argentina; ^3^ Azistia Buenos Aires Argentina; ^4^ Atencion de Pacientes Shire Argentina

**Keywords:** diagnosis, Fabry disease, family tree, pedigree, X‐linked

## Abstract

**Background:**

Fabry disease (FD) is an X‐linked disorder of glycosphingolipid catabolism caused by a deficiency of the lysosomal enzyme alpha‐galactosidase A (*GLA*). FD is still an underdiagnosed disorder worldwide. Moreover, there is delay between symptom onset and Fabry diagnosis of at least 10 years. Family screening offers an important benefit for detection of new patients. The aim of this work is to present the approach along with the results of a targeted genetic strategy for pedigree analysis for FD in Argentina.

**Methods:**

By this strategy as soon as a new index Fabry patient is diagnosed, the pedigree group contacts the physician and a meeting is arranged with the physician and the family to build the family tree.

**Results:**

Pedigree analysis was carried out for full in 31 families. In the work period, we have tested 1,462 relatives, and 501 were diagnosed FD. The proportion of positive detection was 33%.

**Conclusion:**

The targeted family screening approach is successful to detect undiagnosed Fabry patients. By this approach, the highest ratio index to pedigree ever reported for FD pedigree analysis of 1:15 was obtained.

## 1 INTRODUCTION

Fabry disease (FD) (OMIM 301500) is an X‐linked disorder of glycosphingolipid catabolism caused by a deficiency of the lysosomal enzyme alpha‐galactosidase A (*GLA*). This enzymatic defect leads to a chronic and progressive systemic accumulation of glycosphingolipids, mainly globotriaosylceramide, in lysosomes of cells of different tissues. Clinical presentation in classical Fabry hemizygous males may present during childhood or adolescence with acroparesthesia, anhidrosis, angiokeratoma, cornea verticillata, gastrointestinal symptoms, and microalbuminuria. At a later age, progressive renal failure, hypertrophic cardiomyopathy and cerebrovascular disease can occur, reducing life expectancy (Mehta et al., 2004). Most heterozygous women are also affected, but demonstrate a more variable phenotype. Later onset forms of FD also occur, with signs and symptoms usually presenting at an adult age and generally restricted to one main affected organ (Doheny et al., 2018).

Diagnosis of FD patients generally starts by clinical suspicion by physicians based on clinical examination of the patient, his medical and family history. Laboratory confirmatory diagnosis of FD in males is carried out by determination of *GLA* activity in leukocytes, followed by mutation analysis; on the other hand only genetic test is useful for diagnosis in females.

The estimated prevalence is around 1:40,000 to 1:117,000 males (Meikle, Hopwood, Clague, & Carey, 1999; Poorthuis et al., 1999). However, a pilot newborn screening study showed a much higher incidence of 1 of 3,600 (Spada et al., 2006). This difference implies FD is still an underdiagnosed disorder worldwide. Moreover, there is delay between symptom onset and Fabry diagnosis of at least 10 years (Reisin, Perrin, & García‐Pavía, 2017). Other strategies to detect still undiagnosed patients is through at risk screenings (Linthorst et al., 2010) and family screening (Rozenfeld, Ceci, Roa, & Kisinovsky, 2015).

Family screening offers an important benefit for detection of new patients. Diagnosis by family screening has the potential of diagnose relatives at an earlier age as compared as if the patients would have been diagnosed by clinical suspicion.

The aim of this work is to present the approach along with results of a targeted genetic strategy for pedigree analysis for FD in Argentina.

## 2 ACTIVITIES OF THE PEDIGREE GROUP

A program for targeted genetic approach was carried out in order to offer assistance in pedigree analysis to the physicians who have diagnosed a Fabry index case. The study period is from April 2003 to November 2018. The program is carried out by a multidisciplinary group consisting of social worker, geneticist, and biochemist.

As soon as a new index Fabry patient is diagnosed, the pedigree group contacts the physician in order to offer him the assistance by the pedigree program group. After authorization by the physician a meeting is arranged with the group, the physician, and the family. In this meeting the index case or other members of the family are asked to provide information of the family and to build together the family tree.

The first step is to analyze if the mutation in the index case is de novo or inherited. For this task we ask for samples of the parents of the index case. If the mutation is present in the family, we continue analyzing the family tree. As the family tree is being done, the biochemist and geneticist guide doctor on which family member has a risk to be affected due to X‐linked inheritance. The family screening is done step by step, from old members first and then going down in the tree of the positive relatives that are being detected.

In several cases, the family relatives live far away from the index case. The mapping is done by social worker who makes connections, investigates, visit different locations looking for other members of family.

Blood samples were collected by venipuncture. We received dried blood spots from male patients for enzymatic activity assay. If the result is pathologic, 10 ml of heparin blood in order to confirm the diagnosis by measuring *GLA* activity in leukocytes. EDTA blood was received from female patients for genetic test, and also from male Fabry patients diagnosed by deficient *GLA* activity in leukocytes.

The procedures followed were in accordance with the ethical standards of the Ethical Committee of AADELFA (CABA, Argentina) and with the Helsinki Declaration of 1975, as revised in 2013. All patients provided written informed consent to participate in this study.

## 3 ENZYMATIC ACTIVITY DETERMINATION

The *GLA* activity determination was carried out on dried blood filter paper or leukocytes, according to the method of Chamoles, Blanco, and Gaggioli (2001) and described in Ceci et al. (2011).

## 4 MUTATION DETECTION IN *GLA* GENE

Mutation analysis of *GLA* gene (NG_007119.1) was done using DNA isolated from EDTA blood samples. Polymerase chain reaction amplification of specific exon by the use of specific primers was carried out. The amplicons were purified and then sequenced in both directions in a DNA Sequencing device (Applied Biosystems, Foster City, California).

In the period we have worked with 36 different Fabry families. Each family has a different mutation. Type of mutation detected is represented in Table 1. The most frequent are the missense mutations, accounting for 64% of them, followed by deletion and nonsense mutations. Insertions and splicing were the less frequent.

**Table 1:** Type of mutation detected in the 36 families

	Quantity (*n*)	Percentage (%)
Splicing	1	3
Insertion	2	6
Deletion	5	14
Nonsense	5	14
Missense	23	64
	36	100

The first part of the work consisted of analyzing if the mutation is inherited or de novo. In 5 of the families (14%), the mutation was de novo. In this 5 families, pedigree analysis was carried only with the descendants of the index case. In only 1 family (N° 19) there were descendants, we studied 3 daughters from the index case who were heterozygotes.

So, pedigree analysis was carried out for full in 31 families in which the mutation was inherited by the index case. In the work period, we have tested 1,462 relatives, and 501 were diagnosed FD (Table 2). The proportion of positive detection was 33%. The ratio index to pedigree is 1:15, it means for each index case we detected 15 more Fabry patients in average by this pedigree analysis program. This number emphasizes even more the great success of this approach. As can be observed the number of heterozygotes is the double of hemizygotes, which is characteristic due to the X‐linked inheritance of this disorder.

**Table 2:** The table shows the number of female, male and total relatives studied from the 31 families, and the number of positive cases. In this table, the index cases are included in the numbers

Female studied	Female positive	Male studied	Male positive	Total studied	Total positive
895	339	567	162	1,462	501

We obtained a high proportion of positive cases detection. The reasons are numerous:

1. The use of a targeted genetic approach versus at random analysis. Pedigree analysis in this work was done generation by generation in a stepwise mode, analyzing each time the risk of each patient of having inherited the mutation. We have analyzed only at risk cases. In this way we avoided testing relatives who have no risk at all such as children of normal parents or sons from affected males.
2. Pedigree tree: The social worker in the pedigree analysis team had a central role. Her work is to contact the relatives who have a greater knowledge of the family. It let us to build a more complete and expanded tree.
3. Distances: we could contact relatives living far away from the index case in our vast country. For example, in family 4, there were relatives living 3,000 kilometers far away. In other families, there are relatives living in 3 or more different provinces.
4. Assistance to the index case physician: In most of the cases, physicians of the index cases are nephrologists or cardiologists. They do not have experience in genetic diseases and how to carry out pedigree analysis and genetic counseling. Moreover, generally, they do not have enough time in their consultant to work with the family. The assistance of the pedigree team, who works in close contact with him, gives him the possibility to have the whole family diagnosed.

There is no vast information on pedigree analysis in FD. Few experiences of pedigree analysis are reported in literature, with variable ratios between 1:2 and 1:13. In this work, to our knowledge we are reporting the highest ratio of pedigree analysis for FD of 1:15. The ratio of 1:13 was reported in a work with only 2 families (Maron et al., 2018).

In conclusion, the targeted family screening approach is successful to detect undiagnose Fabry patients. By this approach, the highest ratio ever reported of 1:15 was obtained.

## CONFLICT OF INTEREST

PR and MF had received grants and consultant fees from Shire. RN, RF and RC had no conflict of interest. BM and YC are employees of Shire.
